# Longer pregnancy and slower fetal development in women with latent "asymptomatic" toxoplasmosis

**DOI:** 10.1186/1471-2334-7-114

**Published:** 2007-10-04

**Authors:** Šárka Kaňková, Jaroslav Flegr

**Affiliations:** 1 Department of Parasitology, Charles University in Prague, Faculty of Science, Viničná 7, CZ-128 44 Praha 2, Czech Republic

## Abstract

**Background:**

The purpose of this study was to confirm that women with latent toxoplasmosis have developmentally younger fetuses at estimated pregnancy week 16 and to test four exclusive hypotheses that could explain the observed data.

**Methods:**

In the present retrospective cohort study we analysed by the GLM (general linear model) method data from 730 *Toxoplasma*-free and 185 *Toxoplasma*-infected pregnant women.

**Results:**

At pregnancy week 16 estimated from the date of the last menstruation, the mothers with latent toxoplasmosis had developmentally younger fetuses based on ultrasound scan (*P *= 0.014). Pregnancy of *Toxoplasma*-positive compared to *Toxoplasma*-negative women was by about 1.3 days longer, as estimated both from the date of the last menstruation (*P *= 0.015) and by ultrasonography (*P *= 0.025).

**Conclusion:**

The most parsimonious explanation for the observed data is retarded fetal growth during the first weeks of pregnancy in *Toxoplasma*-positive women. The phenomenon was only detectable in multiparous women, suggesting that the immune system may play some role in it.

## Background

Toxoplasmosis, a zoonosis caused by a protozoan, *Toxoplasma gondii*, is probably the most widespread human parasitosis. In immunocompetent humans, postnatally acquired toxoplasmosis is either inapparent, or accompanied by cervical lymphadenopathy with fever, joint pain, headache and tiredness [1,2]. The acute disease promoted by rapidly dividing tachyzoites usually spontaneously proceeds to the latent toxoplasmosis. During latent toxoplasmosis the parasite survives in the form of slowly dividing bradyzoites in tissue cysts, usually providing immunity against reinfection for the rest of the host life. Latent toxoplasmosis is generally considered to be asymptomatic from the clinical point of view; however, it is accompanied by specific changes in personality profiles of the infected subjects [3]. The most damaging form of toxoplasmosis is congenital toxoplasmosis. In pregnant women with the acute form of infection, the parasite can infect the placenta and, after a lag period, also the fetus. The pathology of the infected newborn has been extensively reviewed. The classical Sabin's triad of symptoms of intrauterine toxoplasmosis includes hydrocephalus, intracranial calcification, and chorioretinitis [4]. Approximately 20% of infants born with congenital infection have severe disease. Another about 70% are asymptomatic at birth but can develop clinical signs in the later years, i.e. slower neurological and mental development and late chorioretinitis [5].

For pregnant women with latent toxoplasmosis neither pathological changes in newborns nor health damage due to toxoplasmosis in mothers have been reported. It was speculated about possible effects of latent toxoplasmosis on the risk of abortion [6]; however, this speculation has not been confirmed in recent studies [7,8]. Women infected with parasite *Toxoplasma *have more sons [9]. In a large cross-section study performed on a sample of 1,736 clients of three obstetrics and gynaecology clinics, the sex ratio (the probability of the birth of a boy) increased up to the value of 0.71, which means that women with the highest concentration of anti-*Toxoplasma *antibodies (and therefore probably recent infection) gave birth to 250 boys per 100 girls. Moreover, slower fetal development was observed at estimated pregnancy week 16 in women with latent toxoplasmosis based on ultrasonography [10]. Several explanations for this phenomenon were suggested, including retarded fetal growth in *Toxoplasma*-infected women. The aims of the present study were to confirm the retarded fetal development in an independent retrospective cohort study, and to test four exclusive hypotheses that could explain the observed data:

### Hypothesis 1

In infected women the fetal development is slower but the delivery does not take place until the fetus reaches the usual birth parameters, e.g. birth weight and length. Therefore infected women are likely to have longer pregnancy as estimated both from the date of the last menstruation and based on ultrasonography.

### Hypothesis 2

In infected women, the fetal development is slower but pregnancy duration is still 280 days on average as in *Toxoplasma*-free women independently of the child's size at birth. Therefore infected women are likely to have the same pregnancy length as *Toxoplasma*-free women estimated based on the date of the last menstruation and shorter pregnancy estimated based on the first ultrasonography. Infants of infected women are likely to have lower birth weight.

### Hypothesis 3

In infected women, ovulation occurs in the later phase of the menstruation cycle. Therefore the first ultrasonography shows correctly the fetal age, pregnancy duration estimated based on ultrasonography is the same as in women without toxoplasmosis but the pregnancy duration estimated from the date of the last menstruation is longer.

### Hypothesis 4

Women with and without toxoplasmosis differ from each other in the secondary sex ratio [9] and the reported difference between growth curves for male and female fetuses explains the slower fetal development in infected women at pregnancy week 16. Therefore no differences in fetal age and pregnancy length will be observed between *Toxoplasma*-infected and *Toxoplasma*-free women at pregnancy week 16 when the male and female fetuses are studied separately.

In our cohort study, we analysed clinical records of 1,035 pregnant women tested for the presence of anamnestic antibodies against *Toxoplasma *to decide which of the four above mentioned hypotheses best corresponds to the empirical data.

## Methods

The experimental set consisted of clients of two private clinics (Centres of Reproductive Medicine in Prague 5 and Prague 8). The experimental design was a cohort study. With the help of the personnel from these clinics, anonymous data was collected on the progress of 1,053 pregnancies. The original data set included records of all clients tested for toxoplasmosis from 1996–2004 who were Czech citizens and inhabitants of the city of Prague. Women were serologically tested for toxoplasmosis at about pregnancy week 16. The presence of anamnestic antibodies against *Toxoplasma *was diagnosed with the indirect immunofluorescence test (IIFT) at dilutions between 1:8 and 1:1024. The samples with specific fluorescence visible in a 1:16 or higher dilution was considered as *Toxoplasma*-positive. In the study set, 208 women tested *Toxoplasma*-positive (prevalence of 19.8%). Clinical records comprised maternal antibody titres, age, number of previous deliveries, number of previous abortions, newborn's sex and birth weight and length, date of the last menstruation, pregnancy length estimated based on the first ultrasonography (mostly between pregnancy weeks 8 and 12), and bioparameter data from fetal ultrasonography (biparietal diameter, abdominal circumference, femur length) done approximately at pregnancy weeks 20 and 30. The data on the last menstruation can be affected by stochastic errors; however, stochastic errors cannot be responsible for false positive results.

The average maternal age was 30 (19–44). Thirty-six women gave birth to twins, with 7 of them being *Toxoplasma*-infected. The data set included 642, 342, 58, 10 and 1 records concerning newborns from the first, second, third, fourth and fifth pregnancy, respectively. Data on the twins were excluded from the data set. Given that some records were incomplete, 66 women with missing data were excluded from the respective analyses. For example, some women were excluded because of the missing data on pregnancy length estimated based on ultrasonography at pregnancy week 16 (43 cases), suspected acute toxoplasmosis, i.e. high IgM and IgA titres (2 cases) or probable error in the date of the last menstruation when the difference in pregnancy length estimated based on ultrasonography and from the date of the last menstruation was greater than 20 days (21 cases).

The influence of toxoplasmosis on pregnancy length at pregnancy week 16 estimated based on the first ultrasonography was evaluated by the GLM (general linear model) method. Independent variables in the model were the binary variable TOXO and continuous variables maternal age and pregnancy length estimated from the last menstruation. The GLM model was also used for studying the effects of toxoplasmosis (independent variable TOXO) and maternal age (independent continuous variable), on the newborn's birth length and weight, fetal bioparameters measured by ultrasonography at pregnancy weeks 20 and 30, and pregnancy length. The continuous variable pregnancy length estimated from the date of the last menstruation was included into the model used for testing the effect of toxoplasmosis on fetal bioparameters. Women with preterm delivery (pregnancy length less than 266 days [10]), were excluded from testing for the influence of latent toxoplasmosis on overall pregnancy length estimated both based on ultrasonography and from the date of the last menstruation. All statistical testing including the evaluation of statistical test assumptions (normality of data distribution, normality of residuals and homogeneity of variances) were done using the Statistica^® ^6.0 software. The study was approved by the IRB of the Faculty of Science, Charles University, and complied with the current laws of the Czech Republic.

## Results

**Figure 1:**
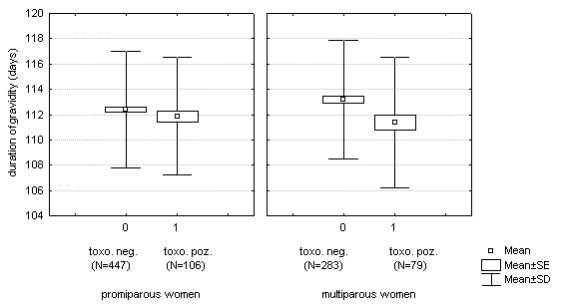
Differences in pregnancy length estimated based on ultrasonography at pregnancy week 16 (estimated from the date of the last menstruation) between *Toxoplasma*-infected and *Toxoplasma*-free women. Left graph, primparas (*P *= 0.2546), right graph, multiparas (*P *= 0.0164).

The influence of toxoplasmosis on pregnancy length at pregnancy week 16 estimated based on the first ultrasonography was evaluated by the GLM in the set of 915 mothers. The result showed that 185 *Toxoplasma*-infected subjects had a shorter pregnancy (111.56 days) than 730 *Toxoplasma*-free subjects (112.59 days) (F_1,911 _= 6.1, *P *= 0.0137). The continuous predictors in the model were also significant: maternal age (F_1,911 _= 4.0, *P *= 0.0456), and presumptive pregnancy length estimated from the date of the last menstruation (F_1,911 _= 484.0, *P *< 0.0001). The same model was used for evaluating the effects of toxoplasmosis separately in women with male and female offspring. In both cases the influence of latent toxoplasmosis on pregnancy length was significant in one-tailed tests (male newborns: n = 507, F_1,503 _= 3.5, *P*_1 _= 0.0313, female newborns: n = 396, F_1,392 _= 2.9, *P*_1 _= 0.0459). The separate tests for primiparous and multiparous women showed that the effect of toxoplasmosis was significant for multiparous women (n = 362, F_1,358 _= 5.8, *P *= 0.0164), while for primiparous women this effect was not significant (n = 553, F_1,549 _= 1.3, *P *= 0.2546) (Figure 1).

Toxoplasmosis had no effect on fetal bioparameters measured by ultrasonography at pregnancy weeks 20 and 30 in both the whole study set (Table 1) and the subset of multiparous women (not shown). Similarly, toxoplasmosis had no effect on newborn's birth length and weight both in the whole study set: birth length (n = 784, F_1,780 _= 1.6, *P *= 0.2071, averages: 50.47 cm in *Toxoplasma*-infected and 50.66 cm in *Toxoplasma*-free women), birth weight (n = 962, F_1,958 _= 0.00, *P *= 0.9872, averages: 3497.61 g in *Toxoplasma*-infected and 3493.44 g in *Toxoplasma*-free women) and in the subset of multiparous women: birth length (n = 324, F_1,320 _= 2.9, *P *= 0.0873, averages: 50.30 cm in *Toxoplasma*-infected and 50.77 cm in *Toxoplasma*-free women), birth weight (n = 398, F_1,394 _= 0.23, *P *= 0.6284, averages: 3499.61 g in *Toxoplasma*-infected and 3535.18 g in *Toxoplasma*-free women).

**Table 1:** Fetal ultrasonography data in *Toxoplasma*-infected and *Toxoplasma*-free women at pregnancy weeks 20 and 30

		20th week of the gravidity			30th week of the gravidity		
		
		BPD	AC	FL	BPD	AC	FL
*n *(toxo.neg./toxo.poz.)		952 (760/192)	925 (378/187)	952 (760/192)	940 (750/190)	913 (727/186)	937 (747/190)
F		0.0	1.4	3.1	0.2	0.0	1.0
*P*		1	0.2432	0.0781	0.6291	0.8679	0.3124
Mean (SE)	toxo. neg.	48.94(0.14)	153.27(0.53)	34.58(0.12)	78.21(0.13)	266.64(0.56)	58.71(0.12)
	toxo. poz.	49.01(0.26)	152.57(0.98)	34.97(0.23)	78.41(0.24)	267.18(1.05)	59.00(0.21)
maternal age	F	12.8	16.2	4.3	24.0	31.5	22.2
	*P*	0.0004	< 0.0001	0.0388	< 0.0001	< 0.0001	< 0.0001
pregnancy length	F	860.2	864.9	687.7	592.0	467.2	589.2
	*P*	< 0.0001	< 0.0001	< 0.0001	< 0.0001	< 0.0001	< 0.0001

Fetal parameters are shown in millimetres. The results of statistical tests (F and *P*) were obtained with toxoplasmosis as the independent variable and maternal age and pregnancy length estimated from the date of the last menstruation as continuous predictors. BPD – biparietal diameter, AC – abdominal circumference, FL – femur length.

**Figure 2:**
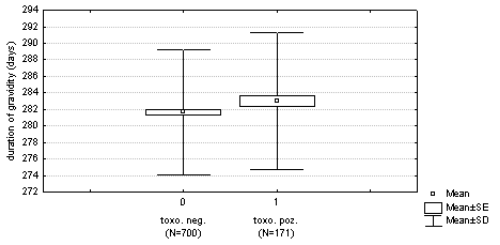
Differences in pregnancy length estimated from the date of the last menstruation between *Toxoplasma*-infected and *Toxoplasma*-free women (*P *= 0.0151).

**Figure 3:**
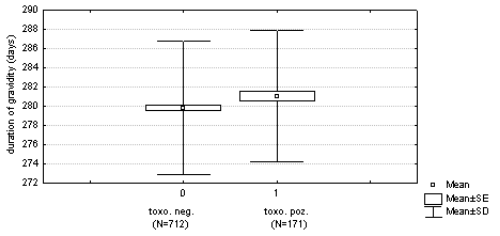
Differences in pregnancy length estimated based on ultrasonography between *Toxoplasma*-infected and *Toxoplasma*-free women (*P *= 0.0256).

*Toxoplasma*-infected women had longer pregnancy than *Toxoplasma*-free women (281.03 vs. 279.82 days, based on ultrasonography) (283.02 vs. 281.65 days, based on the date of the last menstruation) (Figures 2 and 3). The effects of toxoplasmosis and maternal age on pregnancy length were significant (based on ultrasonography: n = 884, F_1,881 _= 5.0, *P *= 0.0256, maternal age: F_1,881 _= 4.3, *P *= 0.0378), (based on the date of the last menstruation: n = 871, F_1,868 _= 5.9, *P *= 0.0151, maternal age: F_1,868 _= 18.0, *P *= 0.0001). To estimate influence of available confounding variables and interactions, we also analysed two models with independent variables TOXO, newborn's SEX and PARITY (0 – primipara, 1 – multipara), and the dependent variable pregnancy length estimated either based on ultrasonography or from the date of the last menstruation. The effect of TOXO on pregnancy length estimated both based on ustrasonography and from the date of last menstruation was significant (pregnancy length^USG^: n = 882, F_1,873 _= 4.9, *P *= 0.0264, maternal age F_1,873 _= 3.9, *P *= 0.0491, newborn's SEX F_1,873 _= 0.9, *P *= 0.3303, PARITY F_1,873 _= 1.5, *P *= 0.199; pregnancy length^LM^: n = 869, F_1,860 _= 6.1, *P *= 0.0138, mother's age F_1,860 _= 16.6, *P *= 0.0001, newborn's SEX F_1,860 _= 0.2, *P *= 0.674, PARITY F_1,860 _= 0.6, *P *= 0.663). On the other hand, no effect of interaction, including TOXO-PARITY and TOXO-newborn's SEX, was significant in any model.

## Discussion

The results confirmed the already reported differences in the early fetal development between *Toxoplasma*-infected and *Toxoplasma*-free women. As for our four proposed hypotheses, the obtained data confirm hypothesis No. 1 that assumes the slower fetal development in women with toxoplasmosis with the determination of pregnancy length based on fetal bioparameters. Hypotheses No. 2 and 3 can be rejected on the basis of the observed differences in pregnancy length between *Toxoplasma*-infected and *Toxoplasma*-free women and Hypothesis No. 4 was disproved on the basis of positive results of the analysis performed separately for the male and female offspring.

The slower fetal development in infected women can be explained at least by three principally different proximal mechanisms. *Toxoplasma *could directly retard the fetal development or could shift the moment of the implantation of the blastocyst or reduce the stringency of embryo quality control, thus allowing fetuses with mild developmental disorders to escape it. Retarded fetal development could be a result of subclinical health impairment in *Toxoplasma*-infected women. Chronic pathogenic processes can affect the circulation of blood in the uterus and maternal part of the placenta with consequent fetal malnutrition [11]. Toxoplasmosis could cause defects in the general metabolism and fetal growth in *Toxoplasma*-infected women. In its early phase, the fetal development is most susceptible to environmental disturbances [12]. It is in agreement with our observation, namely with the fact that the differences between *Toxoplasma*-infected and *Toxoplasma*-free women were strongest early in pregnancy, and turned out to be nonsignificant at pregnancy weeks 20 and 30.

The influence of toxoplasmosis on pregnancy was only observed in multiparous women and remained nonsignificant in primiparous women. It suggests that the maternal immune system could play some role in the proximate mechanism of impairment of the fetal development in *Toxoplasma*-infected women. Immunological memory is suspected to be responsible for the differences between the first pregnancy and the following ones [13,14]. Two different immunological hypotheses can be suggested for the effect of toxoplasmosis on pregnancy. The first one assumes that the changes to the immune system could delay the implantation of the blastocyst in multiparous women with toxoplasmosis. The maternal immunoreactivity against the blastocyst is known to play a significant role in the process of implantation [15]. Toxoplasmosis is accompanied by the production of immunosuppressive lymphokines IL-10 and TGF-beta [16,17] that can interfere with the process of immunization. Therefore, *Toxoplasma*-infected women could be less immunized by antigens on the surface of the blastocyst during the first pregnancy and consequently, the blastocyst implantation in later pregnancies could be delayed.

The other hypothesis suggests that *Toxoplasma *could weaken or switch off the mechanism of spontaneous abortion, normally responsible for removal of embryos with developmental defects (and also with a slower fetal growth rate). Some components of the immune system probably play a role in the effector arm of this quality control process. Toxoplasmosis is known to be associated with suppression of the immune system [18]. Due to immunosuppression, *Toxoplasma*-infected mothers could bear to full term more children with developmental defects. This could explain the smaller fetal size at the first ultrasonography as well as longer pregnancy in *Toxoplasma*-infected women. The stronger effect of toxoplasmosis in multiparous women can result from the previous immunization by trophoblast or embryonic antigens leading to generally higher stringency of quality control mechanisms. In *Toxoplasma*-free multiparous (and therefore immunized) women, most fetuses with any developmental defect are likely to be aborted. In *Toxoplasma*-infected women whose immunization against trophoblast and embryonic antigens is lower (due to *Toxoplasma *induced immunosuppression), a higher percentage of defective embryos are likely to survive.

Most previous studies assumed that the latent toxoplasmosis-associated behavioural and physiological changes are a product of *Toxoplasma *infection [19]. It cannot be excluded by any case-control study, however, that *Toxoplasma *infection is in fact only statistically associated with some unknown socioeconomic or possibly even infection factor that is responsible for the observed effects. On the other hand, most toxoplasmosis-associated effects such as changes in novelty seeking, activity, reaction times and the secondary sex ratio have been already confirmed by experimental infection of laboratory mice or rats [19,20]. Therefore, the direct effects of *Toxoplasma *on the organism of its host seem to be the most parsimonious explanation for the observed phenomena.

The effect of latent toxoplasmosis on pregnancy is rather low. The relatively high statistical significance is partly caused by the large size of the studied sample (n = 915); it must be stressed, however, that the effect size estimated on the basis of the eta squared is less than 1%. The difference in pregnancy length of 1.3 days seems to be of no importance for clinical practice. However, the slightly retarded fetal development could not be the only effect of latent toxoplasmosis on pregnancy. Nothing is known either about the possible influence of latent toxoplasmosis in the mother on the postnatal development of her child. Latent toxoplasmosis affects as many as 84% of women in some populations [21], while the risk of acute toxoplasmosis during pregnancy is usually only 1–2% [22]. Therefore, the impact of latent toxoplasmosis on public health can be in fact even higher than that of acute and congenital toxoplasmosis. In this context, more attention should be paid to the study of effects of this "asymptomatic" form of toxoplasmosis on the prenatal and postnatal development in the future.

## Conclusion

The obtained data confirmed that the fetal development in *Toxoplasma*-infected women is slower, but the delivery does not take place until the fetus reaches the usual birth parameters, e.g. birth weight and length. The maternal immune system probably plays a role in the proximate mechanism of impairment of the fetal development in *Toxoplasma*-infected women.

## Competing interests

The author(s) declare that they have no competing interest.

## Authors' contributions

Both authors contributed equally to this work and read and approved the final manuscript.

## Pre-publication history

The pre-publication history for this paper can be accessed here:


